# Social isolation consequences: lessons from COVID-19 pandemic in a context of dynamic lock-down in Chile

**DOI:** 10.1186/s12889-024-18064-1

**Published:** 2024-02-24

**Authors:** Alessandra Patrono, Stefano Renzetti, Cristian Guerini, Mark Macgowan, Stefanny M Moncada, Donatella Placidi, Maurizio Memo, Roberto G. Lucchini

**Affiliations:** 1 Department of Molecular Medicine e. Translational, v.le Europa 11, 25121 Brescia, Italy; 2 Department of Medical-Surgical Specialties, Radiological Sciences and Public Health, v.le Europa 11, 25121 Brescia, Italy; 3 Robert Stempel College of Public Health & Social Work, School of Social Work, Florida International University, 11200 SW 8th Street, AHC-5 Room 513, Miami, Florida 33199 USA; 4 Departamento de Gobierno, Universidad del Desarrollo, Chile, 7610658 Las Condes, Región Metropolitana Chile; 5 Department of Environmental Health Sciences, School of Public Health, Florida International University, 11200 SW 8th St #500, Miami, FL 33174 USA

**Keywords:** Mental health, Psychosomatic, Young adults, COVID-19

## Abstract

**Background:**

Chile did not adopt general and unified lockdowns for the whole nation but organized itself with dynamic and sometimes irregular lockdowns. These dynamics and consequences of social isolation could be generalized to other contexts of isolation such as those affecting minorities such as immigrants, prisoners, refugees.

**Methods:**

In this study, we investigated the physical and mental health symptoms associated with lifestyle changes due to lockdown among university students in Chile. We examined psychopathological variations in relation to mental health problems in a healthy young population. Our goal was to develop interventions to address these new psychosocial problems in potentially comparable post-pandemic contexts. From May 10th 2021 to June 2th 2021, 420 University students took part in an anonymous survey asking for information on habits and symptoms that emerged during the lockdown in response to the COVID-19 pandemic. Three health outcomes were assessed: digestive disorders; headache; fear of COVID-19. Covariates including conditions and lifestyle during the pandemic, SARS-CoV-2 infections in the family, financial situation and productivity were considered in the analysis.

**Results:**

Participants experienced headache and fear of COVID-19 quite frequently during the lockdown period. More than half of the sample also experienced social isolation. Female gender, sleep quality, memory difficulties, and a change in eating habits resulted associated with an increased risk of health outcomes such as headaches and digestive disorders.

**Conclusions:**

The results of this study fit within an original pandemic context: The results of this study can help identify needs and promote solutions applicable to different contexts. Future interventions should focus on the promotion and implementation of healthy habits focused on sleep hygiene, psychoeducation on the use of mobile devices and gender medicine with the support of healthcare organizations and University.

**Supplementary Information:**

The online version contains supplementary material available at 10.1186/s12889-024-18064-1.

## Introduction

The coronavirus disease (COVID-19) pandemic caused by the Severe Acute Respiratory Syndrome coronavirus 2 (SARS-CoV-2) has posed a major public health threat worldwide [1]. The very rapid spread of the disease has forced the nations to adopt radical emergency plans to combat the epidemic, such as social distancing, closure of schools and workplaces and home quarantine [2]. These contrast measures, together with the uncertainty about the disease spread [3], produced a major impact on people's mental health [4–6].

While governments adopted different disease containment measures, nearly all enacted lockdown policies. In Chile and other countries like Brazil, no structured nationwide lockdown was implemented. Some communities and urban areas declared a mandatory quarantine at different times, but on March 16, 2020, all schools were closed in Chile [7, 8]. The closures were followed by the announcement of closing the national borders on March 18, 2020 and the declaration of a national emergency on the same date, and several interventions were born to contain the epidemic [9]. The government decided on a daily basis whether the restrictions should have been extended to new municipalities, according to a policy of "dynamic quarantines" [10], which reflected the intent of avoiding economic consequences as much as possible. The term dynamic quarantine refers to a type of lockdown that is flexible and organized by areas according to the criteria of number of cases and places in available hospitals, allowing the country not to close down completely.The first measures to control the epidemic were the closure of kindergartens and schools, followed by the closure of national borders [11]. On March 25, 2020, the central Chilean government implemented localized lockdowns at the municipality level. The lockdowns were established by the government based on four criteria defined at the municipal level by the Chilean Ministry of Health: i) the total number of active cases, ii) the increase in the incidence of active cases, iii) the total number of active cases per km^2^, iv) the availability of beds in intensive care units (ICUs) [8].

These quite unpredictable containment measures, together with the fear of the disease and the uncertainties deriving from the pandemic development and its socio-economic consequences, impacted the mental and physical well-being of individuals.

Studies have shown how lockdowns have triggered feelings of loneliness, irritability, restlessness and nervousness in the general population [12]. Among the psychological consequences of social isolation and the pandemic, issues of suicidality and health care costs concerned emerged significantly [13, 14].

In addition, a dynamic and unpredictable lockdown scenario impacting on habits has led to a secondary stress.

Considering how other studies have investigated the repercussions of more structured lockdown and the effects on the well-being of individuals [15, 16], this study can help to better understand the relationship between the intensity and modalities of the lockdown measure and the effect on mental health.

## Materials and methods

### Study population

A total of 5,350 students from the University of Desarrollo, in Santiago de Chile, were invited to participate through the institutional mailing list. The questionnaire was sent to all university students. Initially the questionnaire was dedicated only to three-year degree students, then it was extended to all attending students. An exclusion criterion based on age was applied: subjects over the age of 40 were excluded to maintain a criterion of homogeneity of consequences on the young adult population.

The Ethics Committee of the University of Desarrollo, Santiago de Chile, granted the exemption from its authorization as the data collected were anonymous.

### Enrollment and questionnaire

The survey was designed to identify the potential negative implications of dynamic social isolation and to identify risk and protective factors to draw preventive recommendations and solutions for emerging problems affecting the health of young adults. Data were collected through the Google Forms database anonymously. We focused this investigation on the period of isolation from the beginning of the restrictive measures promulgated on May 10th 2021.

The questionnaire was structured in the form of a self-report and created by a working group that included occupational physicians and psychologists. The investigators balanced trying to use as few items as possible to facilitate administration by gathering an adequate amount of information. A total of 138 questions were asked and the questionnaire took between 8 and 10 minutes to complete.

The questionnaire was structured into eight sections: (1) socio-demographic information, including gender, age, and nationality; (2) having experienced social isolation or not; (3) conditions of social isolation, including information on housing conditions and access to a private garden, hours spent away from home, the use of screen time (time spent in front of television, video games or phone) expressed in hours. The frequency of use was then explored as increased, decreased, or unchanged compared with the pre-pandemic period; (4) lifestyle during the lockdown, including questions on alcohol consumption, cigarette smoking, and eating habits. Sleep quality was assessed through a frequency scale, indicating any changes of increased, decreased, or unchanged. Changes in dietary habits compared with pre-isolation habits; (5) physical and psychosomatic symptoms, including fear of infection. Some items were inspired by the Adult Behavior Checklist (ABCL) [17] for the construction of the questionnaire: the relevance of dealing with topics such as physical health symptoms (digestive problems, headaches and sleep disturbances) is to be evaluated in disturbing conditions for the psycho-physical well-being of individuals. The presence of physical symptoms such as headaches and digestive upsets was assessed by asking the participant to respond to the presence or absence of symptoms with a "yes/no" answer. The presence of sleep disturbances was assessed through the investigation of habitual sleep conditions (Poor; Fair good; Good; Excellent) and sleep conditions since the onset of dynamic social restrictions conditions (Worsened; Same; Improved).; (6) SARS-CoV-2 infection in the household, assessed through questions on having symptoms or being positive for COVID-19 as well as information on the death of family members due to COVID-19; (7) economic and financial situation, to assess potential concerns about the possibility of continue studying; and (8) productivity, assessed as a perception of learning and concentration difficulties.

### Statistical analysis

The median as well as the first and third quartiles were used to describe the continuous variables because of their skewed distribution. The absolute and relative frequencies were applied to the categorical variables. Physical and mental health were assessed considering three different outcomes: digestive disorders; headache and fear of COVID-19. Various covariates were considered in the analysis based on the hypothesis that they could have had an effect on the outcomes and on previous studies [18, 19]: the housing conditions during lockdown; the possibility of leaving the house during the day; the lifestyles of the participants as potential protective or harmful factors.

An exploratory factor analysis was first applied to 18 variables in the questionnaire to reduce the number of covariates throughout the definition of latent variables. We then applied a polychoric correlation matrix because of the categorical nature of the variables. The Bartlett’s test of sphericity, the Kaiser–Meyer–Olkin (KMO) criterion and the Cronbach’s alpha were used to assess the suitability of the data for the exploratory factor analysis. A label was attributed to each factor based on the loadings associated with each original variable: the threshold for factor loadings was set to 0.3 for inclusion in a factor. Finally, logistic and ordinal logistic regression models (depending on whether the dependent variable was dichotomous or with more ordinal categories, respectively) were considered to evaluate the impact of the latent variables defined through the factor analysis as well as the socio-demographic data (age, sex), having experienced SARS-CoV-2 infection or social isolation, sleep quality during lockdown and the variables that were not considered suitable for the factor analysis. An interaction term between sex and each covariate was introduced in the regression models to test for a difference between males and females in the association between each variable and the three outcomes. The Tukey method was applied to adjust *p*-values and 95% CI to account for multiple comparisons. A statistical significance level was set at *p* < 0.05. The data were analyzed through the statistical program R (v. 4.3.2).

## Results

### Descriptive statistics

A total of 420 students (7.85% of the invited students at the University of Desarrollo, Santiago de Chile) participated in the survey. Most of the respondents were females (61.4%) with a median age of 21.0 years (1st quartile [Q1], 3rd quartile [Q3] 18, 40), mostly of Chilean nationality (97.1%) (Table 1).

**Table 1:** Descriptive statistics related to the socio-demographic information of the overall 420 participants in the survey for the variables included in the factor analysis and in the final models as covariates

	**Overall (*N*=420)**
**Age**	
Mean (SD)	21.7 (2.7)
Median (Q1, Q3)	21.0 (20.0, 23.0)
**Sex**	
F	258 (61.4%)
M	162 (38.6%)
**Nationality**	
Chilean	408 (97.1%)
Colombian	3 (0.7%)
Paraguayan	1 (0.2%)
Peruvian	1 (0.2%)
Venezuelan	1 (0.2%)
Mexican	2 (0.5%)
Spanish	1 (0.2%)
North American	1 (0.2%)
Other	2 (0.5%)

During the period of dynamic restrictions most of the students (76.4%) lived in a house with 3 to 6 rooms and with a private garden (74.0%) and half of the students shared a home with more than 3 people (50.7%) and spent time away from home on average.

The lifestyle questions revealed that most of the target population did not smoke before and during the lockdown (69.9%)_;_ 12.4% of the interviewees increased their smoking habits. Most of those who habitually consumed alcohol had reduced their consumption (48.1%). 20.7% of those interviewed decreased or worsened their habits related to physical activity (Table 2).

**Table 2:** Descriptive statistics related to social isolation of the overall 420 participants in the survey for the variables included in the factor analysis and in the final models as covariates

	**Overall (*N*=420)**
**Social isolation**	
No	131 (31.2%)
Yes	289 (68.8%)
**Private garden availability**	
No	109 (26.0%)
Yes	311 (74.0%)
**Number of Cohabitants**	
0-1	54 (12.9%)
2-3	153 (36.4%)
>3	213 (50.7%)
**Number of rooms in the house**	
<3	60 (14.3%)
3-6	321 (76.4%)
>6	39 (9.3%)
**Hours spent outside on daily basis**	
0	77 (18.3%)
>0	343 (81.7%)
**Average hours spent using phone**	
<=3 hours	65 (15.5%)
4-6 hours	153 (36.4%)
>6 hours	202 (48.1%)
**Change in time using phone**	
Same/Decreased	71 (16.9%)
Increased	349 (83.1%)
**Average hours spent using PC**	
<=6 hours	115 (27.4%)
>6 hours	305 (72.6%)
**Change in time using PC**	
Same/Decreased	33 (7.9%)
Increased	387 (92.1%)
**Average hours spent playing videogames**	
Never	209 (49.8%)
<1 hour	83 (19.8%)
>=1 hour	128 (30.5%)
**Change in time playing videogames**	
Same/Decreased	337 (80.2%)
Increased	83 (19.8%)
**Average hours spent watching TV**	
Never	119 (28.3%)
<1 hour	140 (33.3%)
>1 hour	161 (38.3%)
**Change in time watching TV**	
Same/Decreased	329 (78.3%)
Increased	91 (21.7%)
**Smoking status during lockdown**	
Non-smoker	293 (69.9%)
Decreased	30 (7.2%)
Same	44 (10.5%)
Increased	52 (12.4%)
**Alcohol consumption during lockdown**	
Never	106 (25.4%)
Decreased	138 (33.1%)
Same	115 (27.6%)
Increased	58 (13.9%)
**Physical activity**	
No	182 (44.3%)
Decreased	85 (20.7%)
Same	36 (8.8%)
Increased	108 (26.3%)
**Usual sleep quality**	
Poor	76 (19.0%)
Fair good	131 (32.8%)
Good	169 (42.4%)
Excellent	23 (5.8%)
**Sleep quality**	
Worsened	253 (63.4%)
Same	122 (30.6%)
Improved	24 (6.0%)
**BMI**	
Mean (SD)	0.0 (0.1)
Median (Q1, Q3)	0.0 (0.0, 0.0)
**Change in weight**	
Lose weight	98 (24.6%)
No	102 (25.6%)
Gained weight	199 (49.9%)
**Change in nutrition**	
Worsened	108 (27.1%)
No	165 (41.4%)
Improved	126 (31.6%)
**Positive to COVID-19**	
No/Don’t know	237 (56.4%)
Yes	183 (43.6%)
**Covid symptoms**	
No	294 (73.7%)
Yes	105 (26.3%)
**Relatives infected by COVID-19**	
No	140 (33.3%)
Yes	280 (66.7%)
**Relatives with COVID-19 symptoms**	
No	135 (32.1%)
Yes	285 (67.9%)
**Relatives died for COVID-19**	
No	351 (83.6%)
Yes	69 (16.4%)
**Cohabitant unemployed because of the pandemic**	
No	266 (71.7%)
Yes	105 (28.3%)
**Mnemonic difficulties**	
No	108 (29.1%)
Yes	263 (70.9%)
**Performance reduction**	
No	169 (45.6%)
Yes	202 (54.4%)

The incidence of symptoms representing the health outcomes observed during the period of the dynamic lockdown ranged between 32.1% for digestive disorders and 86.0% for headaches. The fear of being infected with COVID-19 was high for 44.3% of the students who answered the questionnaire (Table 3).

**Table 3:** Descriptive statistics of the three outcomes: digestive disorders, headache incidence and fear to be infected by SARS-CoV-2

	**Overall (*N*=420)**
**Digestive disorders**	
No	271 (67.9%)
Yes	128 (32.1%)
**Headache**	
No	56 (14.0%)
Yes	343 (86.0%)
**Fear to be infected by SARS-CoV-2**	
Low	85 (20.2%)
Medium	149 (35.5%)
High	186 (44.3%)

### Inferential data analysis

#### Factor analysis

We excluded from the exploratory factor analysis the variables time spent outside, change in time using phone, smoking status during lockdown, alcohol consumption during lockdown and having experienced COVID-19 symptoms since they showed either a KMO index below 0.5 or showed a low internal consistency with a Cronbach’s alpha below 0.5. Bartlett’s test of sphericity (*p* < 0.001) and the KMO criterion (overall KMO value: 0.6) revealed that the data were suitable for an exploratory factor analysis. The factor analysis allowed us to group the remaining 13 variables examined in the questionnaire into the following 5 latent variables (Table 4): Relatives COVID-19 positive, with symptoms or dead; Lockdown conditions (garden availability, having cohabitants, and a higher number of rooms); Mnemonic difficulties and performance reduction; TV usage; Increased physical activity, lost weight and improved nutrition. The coefficient alphas of the internal consistency reliability of the measures ranged between 0.51 and 0.7. The loadings estimated by the factor analysis are shown in Figure S1. The change in weight showed a negative weight, meaning that a decrease in weight during lockdown contributed to higher values of the latent variable related to increased physical activity, lost weight and improved nutrition.

**Table 4:** Collected variables, latent variables and the coefficient alphas of the internal consistency reliability (Cronbach’s alpha)

**Original variables**	**Latent variable**	**Cronbach’s alpha**
Relatives positive to COVID-19; Relatives with COVID-19 symptoms; Relatives died for COVID-19	Relatives COVID-19 positives, with symptoms or dead	0.64
Number of rooms in the house; Private garden availability; Cohabitants	Lockdown conditions (garden availability, having cohabitants, and a higher number of rooms)	0.7
Average hours spent using TV; Change in time using TV	TV usage	0.61
Mnemonic difficulties; Performance reduction	Mnemonic difficulties and performance reduction	0.6
Physical activity; Change in weight; Change in nutrition	Increased physical activity, lost weight and improved nutrition	0.51

#### Association between health outcomes and covariates

The information regarding age, sex, SARS-CoV-2 positivity, social isolation, sleep quality during lockdown, the time spent outside, change in time using phone, smoking status during lockdown, alcohol consumption during lockdown and having experienced COVID-19 symptoms was included in the regression models as a covariate to account for its effect on the outcomes.

The logistic regression model showed that age was negatively associated with headache (OR 0.87; 95% CI 0.78, 0.97). Males showed a lower risk of digestive disorders (OR 0.29; 95% CI 0.16, 0.49).

**Fig. 1:**
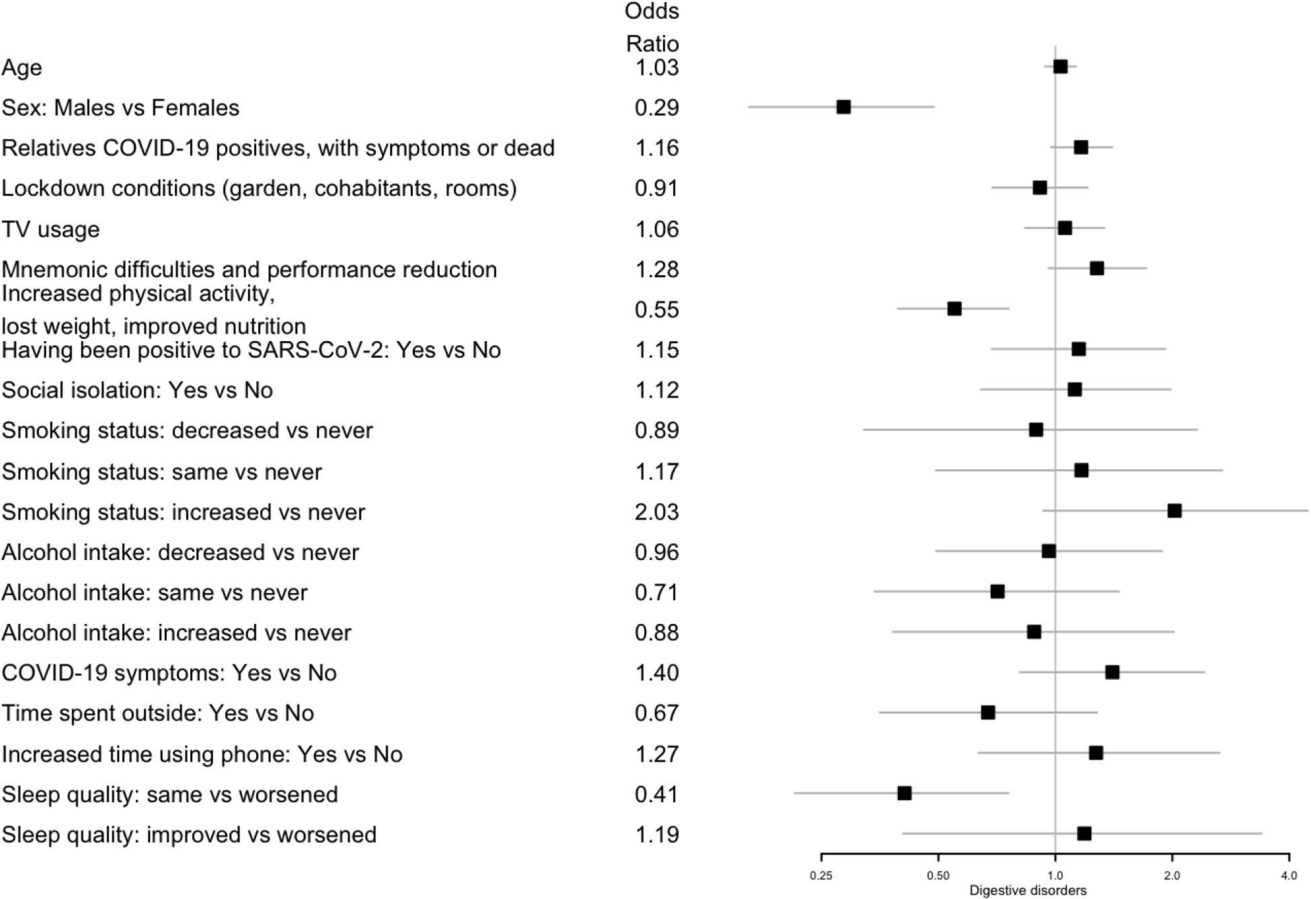
Forest plots for the effect of the covariates on digestive disorders. The squares represent the odds ratios and the lines depict the confidence intervals estimated by the logistic regression

**Fig. 2:**
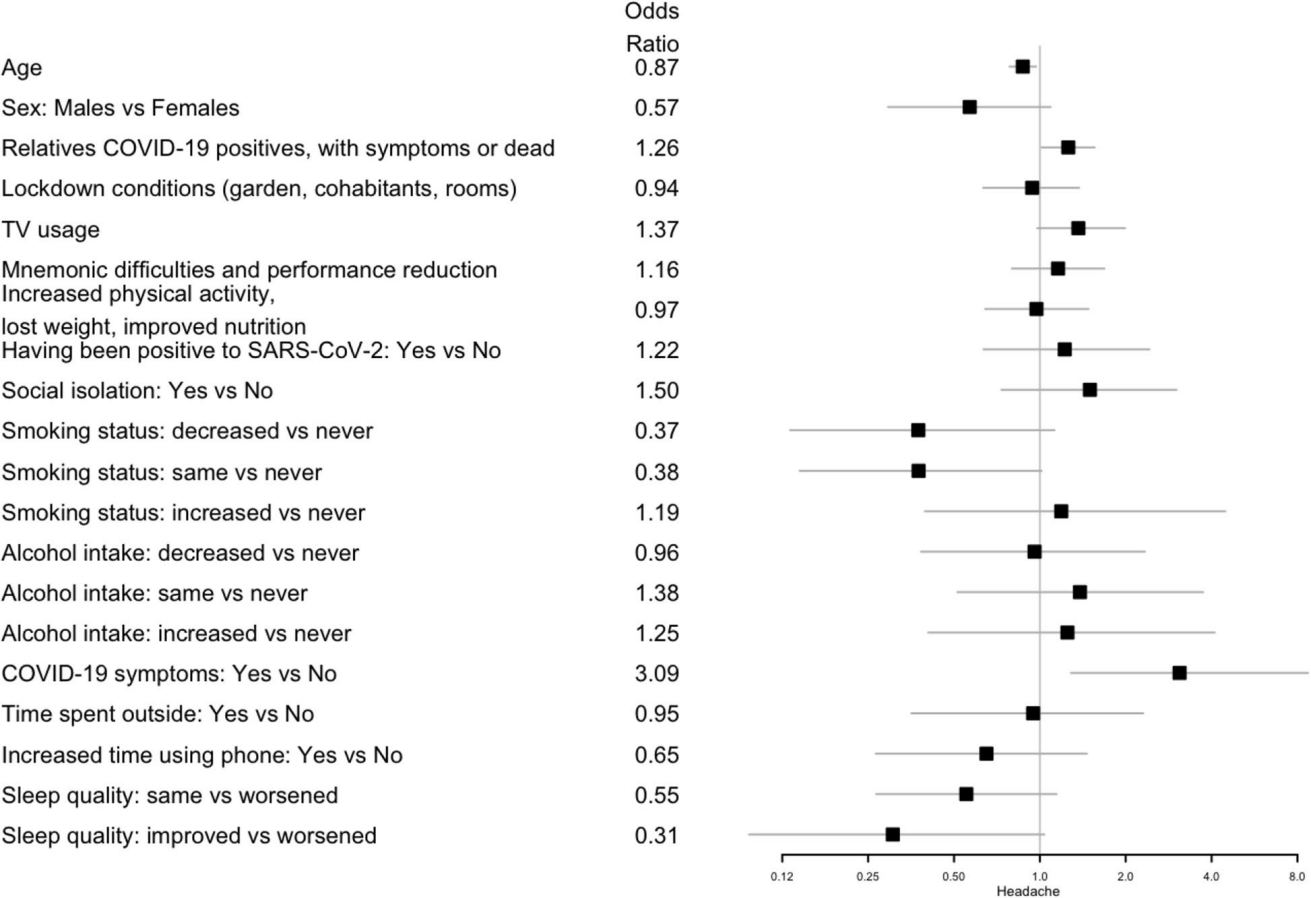
Forest plots for the effect of the covariates on headaches. The squares represent the odds ratios and the lines depict the confidence intervals estimated by the logistic regression

**Fig.3:**
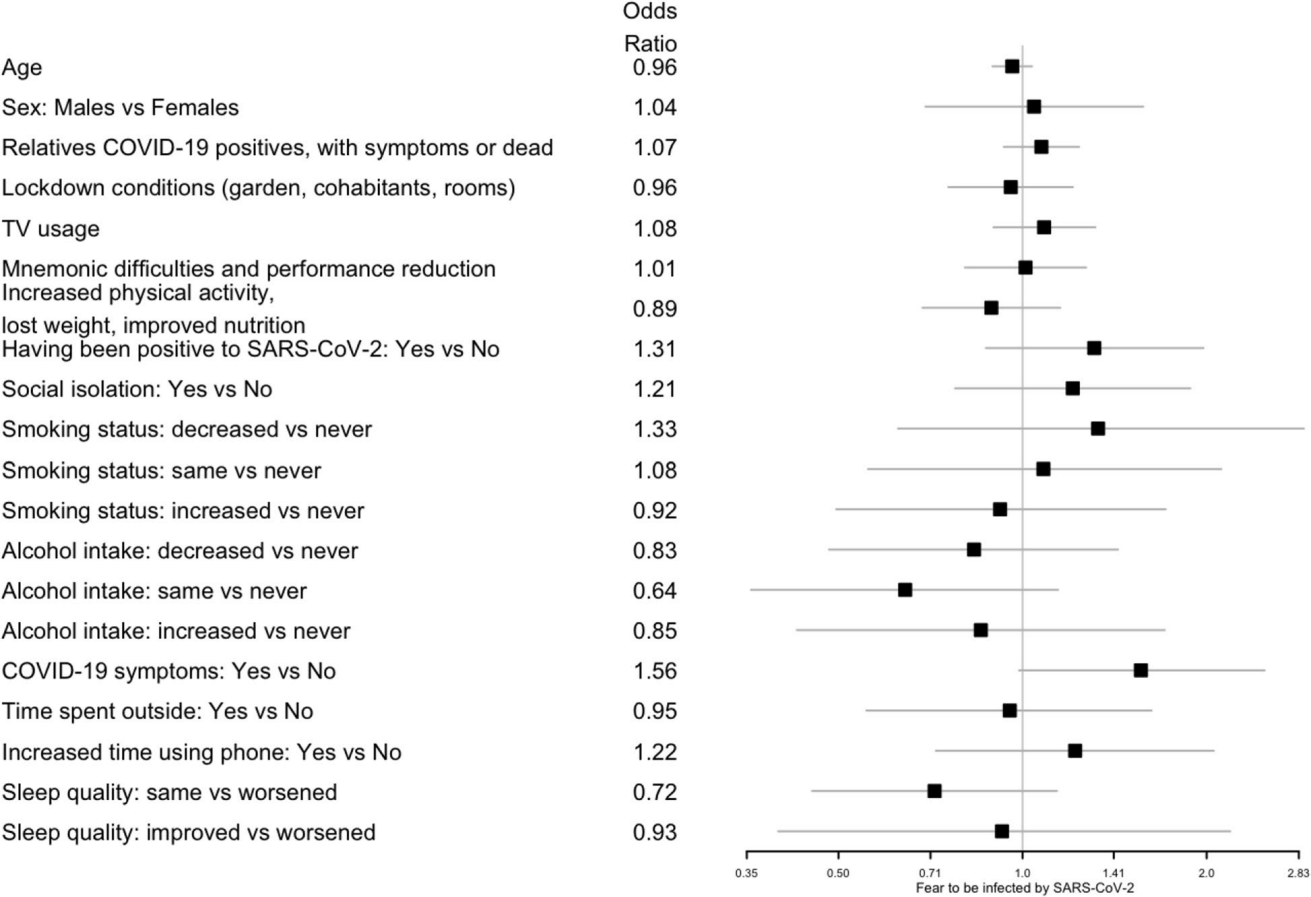
Forest plots for the effect of the covariates on fear of being infected by SARS-CoV-2. The squares represent the odds ratios and the lines depict the confidence intervals estimated by the ordinal logistic regression

Participants that declared to have had relatives COVID-19 positives, with symptoms or dead showed a higher risk of headache (OR 1.26; 95% CI 1.02, 1.56). Poor sleep quality, mnemonic difficulties, and performance reduction were associated with a higher probability of having digestive disorders (OR 1.73; 95% CI 1.29, 2.37) and headache (OR 1.65; 95% CI 1.17, 2.35). Increased physical activity, lost weight and improved nutrition were associated with a lower risk of digestive disorders (OR 0.55; 95% CI 0.39, 0.76). Having had COVID-19 symptoms was associated with higher probability of having headache (OR 3.09; 95% CI 1.29, 8.71). Finally those subjects that declared that sleep quality was the same during the pandemic showed a lower risk of digestive disorders (OR 0.41; 95% CI 0.21, 0.76) while those that experienced an improved sleep quality showed a lower probability of headache (OR 0.31; 95% CI 0.10, 0.99). All the results are summarized in Fig. 1 for digestive disorder; Fig. 2 for headaches; Fig. 3 for fear of being infected by SARS-CoV-2 and in Table S1.

**Fig. 4:**
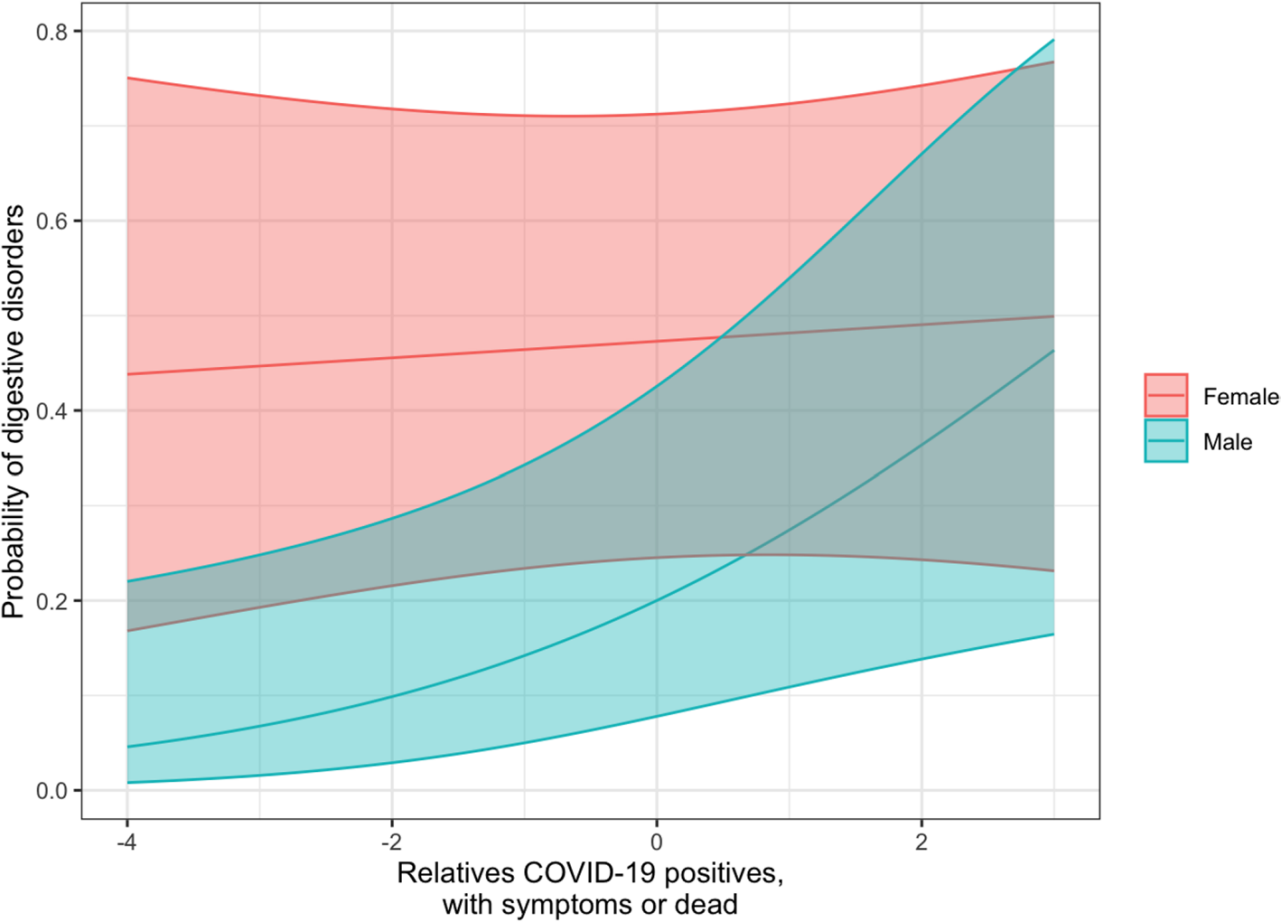
Association between the latent variable “relatives COVID-19 positive, with symptoms or dead” and the probability of having digestive disorders for males and females. The coloured area represents the estimated 95% confidence intervals of the two curves

When an interaction term between each covariate and sex was introduced in the regression models, we only found a marginally significant difference between males and females in the association between relatives COVID-19 positives, with symptoms or dead and digestive disorders (OR 1.459; 95% CI 0.982, 2.168) showing a higher increase of digestive disorders among males (OR 1.511; 95% CI 1.072, 2.131) (Fig. 4 and Table S2).

## Discussion

The aim of this study was to investigate psychosomatic symptoms such as digestive disorders, headache and fear of COVID-19 associated with lifestyle changes due to the COVID-19 pandemic and dynamic and unpredictable social restrictions measures in Chile. In-depth analysis of these issues can help to study potentially comparable contexts of vulnerability and isolation, even post-pandemic.

The target population was composed of university students attending the University of Desarrollo, Santiago de Chile. Participants to the survey reported to have experienced headache, digestive disorders and high fear of getting infected by SARS-Cov-2 during lockdown and pandemic. All of these factors were significantly associated with lifestyle changes, such as nutrition, weight changes, physical activity and the presence of COVID-19 symptoms. Additionally, sleep quality, mnemonic difficulties and performance reduction were found to be associated with digestive disorders and headache.

This survey confirms a gender susceptibility to psychosomatic outcomes such as digestive disorders. Previous studies had in fact demonstrated how vulnerability to stress involved much more the female gender than males [20, 21]. Surveys of the general population have shown that compared to men, women are more likely to report temporary and persistent pain, particularly more severe pain, more frequent pain, and long-lasting pain [22]. Recently, neuroimaging studies showed sex-related differences in brain response to visceral and psychological symptoms, which cannot be explained by sensory input differences. Looking at the brain it is possible to see an inhibition of limbic regions, such as the amygdala when men are experiencing gastrointestinal symptoms; in the same condition, females tend to show greater activation of affective and autonomic areas, like ventromedial prefrontal cortex, amygdala and inferior cingulate [23]. This brain activity pattern could be related to a different experience of gastrointestinal symptoms following stressful situations, which could explain the different report of digestive disorders in our sample between males and females. Our survey, which confirms a trend of this type, follows the need to provide gender-based medicine and care [24]. Gender-specific healthcare will need to address clinical-epidemiological aspects and education of the entire healthcare ecosystem. This could be increased by incorporating these strategies into medical curricula relating to the education, training and professionalism of operators [25]. The use of artificial intelligence in the analysis of big data in public health can also help guide and analyze gender differences to guide targeted treatments [26]. This study found that physical activity, diet, weight changes and symptoms of COVID-19 are associated with digestive disorders. Social isolation reduced the time spent outdoors and physical activities, leading to weight gain. In fact, 49.9% of students reported gaining weight and 27.1% worsening their eating habits. This trend can be associated by the immediate access to food during the quarantine and an increase in the number of snacks during the day, especially for women [27]. However, a trend reversal was seen in 24.6% of respondents who experienced weight loss. This could stem from concerns about weight gain due to disruption of sporting opportunities (thereby reducing caloric intake) or from increased physical activity at home. Exposure to stress can alter both the quantity and quality of caloric consumption [27] and stressful events such as the pandemic can lead to activation of the hypothalamic-pituitary-adrenal (HPA) neuroendocrine axis and increased glucocorticoid synthesis [28]. Triggering behaviors of this type can lead to non-adherence to a varied and healthy diet, leading to gastrointestinal distress [29] and headache [30]. These trends should be prevented in the university settings, by greater promotion of nutritional health courses and access to dietary services.

Among the factors that have contributed to an impact on digestive disorders there have also been changes in lifestyle habits. For example, increased telephone use and poor quality sleep have been associated with worsening or onset of digestive disorders. An increase in phone usage of more than 6 hours of screen time per day was found in almost half of the sample. The relationship between phone use and digestive upset may be multidirectional. In fact, people often adopt unhealthy eating habits while using the phone (e.g. eating snacks, drinks, irregular meals) [31, 32], leading to an increase iof gastrointestinal disorders [33].

Furthermore, during the covid-19 pandemic, one of the main sources of information for university students was social media or the internet [34, 35]. A great deal of information may have been found online, which included misinformation and conspiracy theories. This large amount of information that should be filtered and processed quickly, coupled with the variable nature of information associated with dynamic blocking mode, can foster feelings of uncertainty. All these factors could cause an information overload in the population, which consequently resulted in an increase of stress levels, depression and anxiety [36] manifesting at a psychosomatic level with digestive disorders. However, the relationship between phone use and psychosomatic disorders is two-way: the combination of high amounts of stress and self-doubt has in turn been associated with a higher risk of cell phone addiction [37]. The COVID-19 infodemic requires spending more time with a smartphone and online sources to keep properly updated, increasing the stress and insecurity that lead to cell phone addiction, and therefore creating a vicious cycle. Self-reported stress, depression and anxiety have been associated with gastrointestinal disorders [38]. Therefore this association of increased time in front of the screen and increased discomfort could indirectly explain the higher prevalence of digestive disorders and our study confirms this trend by focusing on the need for psychoeducation courses for the correct use of electronic devices and information. In addition to digestive complaints, the clinical outcome of headache was also observed in 86% of respondents. This is consistent with other studies that have shown headache to be a clinically significant problem during lockdown [39, 40]. However, the relationship between isolation and headache remains controversial: other studies have identified an improvement in symptoms during the pandemic among patients already suffering from severe migraine [41]. Regarding the worsening trajectory of headache, we could identify several contributing factors: social isolation [42], changing sleep habits [43] and eating habits [44]. Changes and obstacles in medical care (basic and specialist) may have contributed to the consolidation of this clinical outcome in a problematic direction [45, 46]. In this study age was also negatively associated with the onset of headache episodes. The age variability of the participants in this study is not large enough to allow inferences related to changes in headache and migraine over the years, but we can hypothesize that younger students were less exposed to "awareness". The recipients of this questionnaire were in fact university students, therefore with a high level of education and previous surveys had highlighted how this could represent a factor of vulnerability as a high level of education could lead to greater awareness and consequently anguish [21] and psychosomatic symptoms such as headache.

Poor sleep quality, memory difficulties and reduced performance increased the risk of developing two of the symptoms examined, headache and digestive disorders. The relationship between sleep and physical disorders such as headache [47] and gastrointestinal disorders [48] has already been widely described in the literature, as well as the link between epidemics and chronic sleep disorders [49] and our investigation confirms these difficulties also in a dynamic and unpredictable context in terms of restrictions. The disruption of sleep quality and quantity has a negative impact on memory performance, furthermore it could affect other aspects such as mental health and quality of life [50].

In general, fear of COVID-19 and stress have been associated with depression [51], so future follow up studies should investigate the level of depression in this population to see if this association is also consistent in a context such as Chile.

This study provides a better understanding of the impact of pandemics on the psychological and physical health of students in a country where lockdown measures were not managed at the national level, but at the community level. While not all participants may have experienced social isolation, overall headache, indigestion and fear of covid appear to be common outcomes for young adults [18]. All of these health outcomes appear to be strongly influenced by decreased physical activity and worsening nutrition, so preventive interventions should be undertaken to maintain these healthy behaviors also when outdoor activity is limited. Sleep quality has an impact on headache and digestive upset, so promoting sleep hygiene practices will improve students’ health. The "dynamic quarantine" policy adopted in Chile, may have created a sense of insecurity and uncertainty in the population. This insecurity can explain the high fear ratios of covid that we have registered in almost half of the students. This aspect could contribute to increased levels of stress and symptoms of anxiety and depression [51]. Fear of the physical consequences caused by the disease, of being a vector of contagion for others, and uncertainty about the future developments may have impacted the study population, negatively influencing the psychophysical well-being of the students. Psychoeducation and teletherapy interventions can be useful to improve the students’ mental health [52]. Wider use of telehealth and teletherapy can help address mental health conditions [53, 54]. Affordable remote medical and mental health care can provide early diagnosis to minimize the severity of the effects of isolation and can have direct consequences for a reduction in school dropouts [55]. It has been shown that mindfulness-based psychological interventions have demonstrated a significant improvement in the feeling of loneliness that could emerge in these situations [56] and this should be taken into account in programs to promote well-being in the university contest [57].

Poor accessibility to health systems [58], poor nutrition [59], isolated conditions [60], concerns about economic factors [61] and poor quality of sleep [62] are all problems that concern not only pandemic events but also some realities of minorities. The findings from this study should be addressed by public health professionals as they can be generalized to other contexts leading to social isolation that were not specifically considered in our study. The situations in which the problem of isolation and forced marginalization arises in potentially healthy populations concern in particular refugees, immigrants, and prisoners. Risk factors such as racism, discrimination, language barriers and separation from family members predispose immigrants and refugees to an increased risk of social isolation and the occurrence of these clinical outcomes without adequate care. This is becoming especially real in the Chilean context, as Chile is increasingly becoming a host country for South-South migration [63].

Another context in which enforced social isolation becomes very significant is prison. Prison is an acute form of social exclusion [64] which does not allow access to key areas of social life [65]. An analysis on a sample of Chilean inmates identified the stress and loss of autonomy due to the prison regime as factors that negatively affect mental well-being [66]. Lack of autonomy and consequent stress are factors that have characterized the pandemic and restriction scenario. In the contexts of social isolation of the prison, other elements also follow the critical elements of the lock-down due to the pandemic: limitations in contact with the outside world, lack of freedom of movement [67]; lack of access to consumer services and deprivation of time management opportunities and deprivation of a sense of security [68].

These elements of continuity between the pandemic scenario that has affected everyone in a dynamic way in Chile and contexts of vulnerability affecting minorities should help understanding the mechanisms useful for preventing clinically significant consequences that also have a negative impact on the costs of health systems.

## Conclusions

Our results have highlighted how changes in lifestyle habits and consequent emergence of clinical outcomes can be accelerated by conditions of physical and social isolation in a dynamic and unpredictable context. Changes in lifestyle habits, stress and gender vulnerability have been shown to be activators of clinical outcomes such as digestive disorders and headache in a population of highly educated young adults. These results should reflect the need to implement interventions of a preventive or remedial nature also in contexts that are potentially similar in terms of vulnerability catalysts.

## Limitations and further research

The sample is relatively small and only featured students affected by the effects of the pandemic may have responded. Despite this, the survey helps to reflect on the consequences of a type of lockdown that differed from other more generalized and structured. Subsequent follow-up investigations may help clarify the long-term impacts of these effects. Given the complexity of psychological and behavioral dynamics, future research could integrate a qualitative evaluation of the psycho-somatic consequences of a collectively traumatic event. Future studies also should use the important lessons learned from the pandemic scenario and deepen the continuity and similarity with contexts of social isolation of minorities to study targeted prevention and containment protocols of clinical and psychosomatic outcomes.

### Supplementary Information

**Supplementary Material 1.**

## Data Availability

The data that support the findings of this study are available on request from the corresponding author, [A.P].
