# Supplementary material for: Social isolation consequences: lessons from COVID-19 pandemic in a context of dynamic lock-down in Chile
Source: BMC Public Health. 2024 Feb 24;24:599. doi: 10.1186/s12889-024-18064-1 (PMC10893693; doi:10.1186/s12889-024-18064-1)
Supplement: Supplementary file 1 — Supplementary Material 1. [file 12889_2024_18064_MOESM1_ESM.docx]

Figure S1. Heatmap of the loadings estimated by the factor analysis. Values below 0.31 were set to 0. The factors represented on the y-axis are: F1=Relatives COVID-19 positives, with symptoms or dead, F2=Lockdown conditions (garden, cohabitants, rooms), F3=TV usage, F4=Mnemonic difficulties and performance reduction, F5=Increased physical activity, lost weight and improved nutrition. The variables displayed on the x-axis are: V1=Number of rooms in the house, V2=Private garden availability, V3=Cohabitants, V4=Average hours spent using TV, V5=Change in time using TV, V6=Physical activity, V7=Change in weight, V8=Change in nutrition, V9=Relatives positive to COVID-19, V10=Relatives with COVID-19 symptoms, V11=Relatives died for COVID-19, V12=Mnemonic difficulties, V13=Performance reduction.


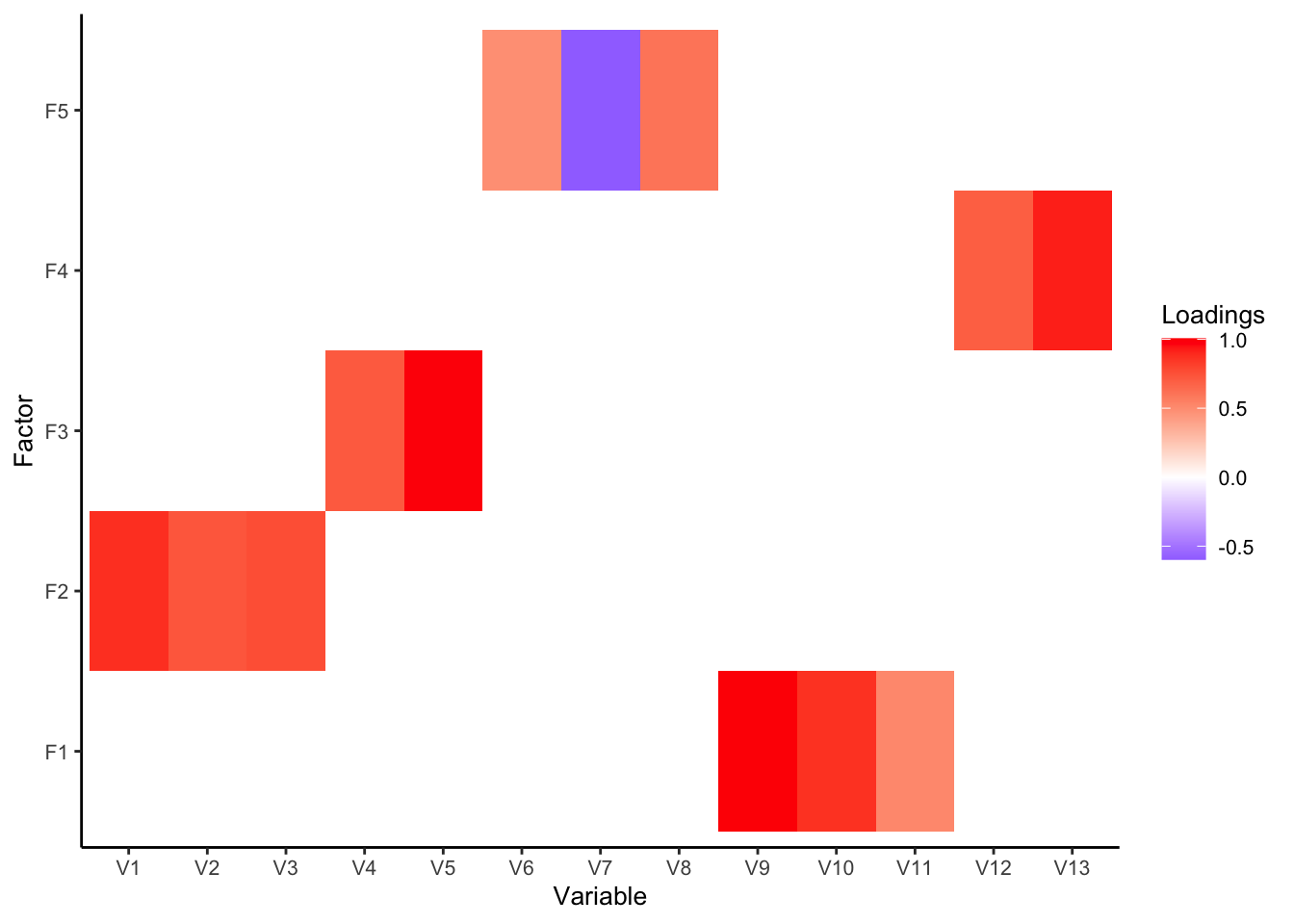


Table S1. Results from the logistic regression (digestive disorders and headache) and ordinal logistic regression (COVID-19 fear) to test the associations between covariates and digestive disorders, headache and fear of COVID-19. The odds ratios (OR), 95% Confidence Intervals (CI) and p-values are shown.

|  | **Digestive disorders** | | | **Headache** | | | **COVID-19 fear** | | |
| --- | --- | --- | --- | --- | --- | --- | --- | --- | --- |
| *Predictors* | *OR* | *95% CI* | *p* | *OR* | *95% CI* | *p* | *OR* | *95% CI* | *p* |
| Age | 1.03 | 0.94 – 1.13 | 0.511 | 0.87 | 0.78 – 0.97 | **0.011** | 0.96 | 0.89 – 1.04 | 0.306 |
| Sex: Males vs Females | 0.29 | 0.16 – 0.49 | **<0.001** | 0.57 | 0.29 – 1.09 | 0.089 | 1.04 | 0.69 – 1.58 | 0.836 |
| Relatives COVID-19 positives, with symptoms or dead | 1.16 | 0.97 – 1.40 | 0.099 | 1.26 | 1.02 – 1.56 | **0.034** | 1.07 | 0.93 – 1.24 | 0.327 |
| Lockdown conditions (garden, cohabitants, rooms) | 0.91 | 0.69 – 1.21 | 0.524 | 0.94 | 0.63 – 1.37 | 0.753 | 0.96 | 0.76 – 1.21 | 0.710 |
| TV usage | 1.06 | 0.83 – 1.34 | 0.634 | 1.37 | 0.98 – 1.99 | 0.083 | 1.08 | 0.90 – 1.32 | 0.407 |
| Mnemonic difficulties and performance reduction | 1.28 | 0.96 – 1.71 | 0.094 | 1.16 | 0.80 – 1.68 | 0.439 | 1.01 | 0.80 – 1.27 | 0.923 |
| Increased physical activity, lost weight, improved nutrition | 0.55 | 0.39 – 0.76 | **<0.001** | 0.97 | 0.64 – 1.48 | 0.897 | 0.89 | 0.69 – 1.15 | 0.374 |
| Having been positive to SARS-CoV-2: Yes vs No | 1.15 | 0.69 – 1.92 | 0.596 | 1.22 | 0.63 – 2.42 | 0.555 | 1.31 | 0.87 – 1.98 | 0.197 |
| Social isolation: Yes vs No | 1.12 | 0.64 – 1.98 | 0.687 | 1.50 | 0.73 – 3.01 | 0.260 | 1.21 | 0.78 – 1.88 | 0.403 |
| Smoking status: decreased vs non-smoker | 0.89 | 0.32 – 2.31 | 0.819 | 0.37 | 0.13 – 1.12 | 0.069 | 1.33 | 0.63 – 2.88 | 0.464 |
| Smoking status: same vs non-smoker | 1.17 | 0.49 – 2.69 | 0.719 | 0.38 | 0.14 – 1.01 | **0.051** | 1.08 | 0.56 – 2.11 | 0.817 |
| Smoking status: increased vs non-smoker | 2.03 | 0.93 – 4.47 | 0.076 | 1.19 | 0.40 – 4.47 | 0.775 | 0.92 | 0.50 – 1.71 | 0.789 |
| Alcohol intake: decreased vs never | 0.96 | 0.49 – 1.88 | 0.910 | 0.96 | 0.38 – 2.33 | 0.926 | 0.83 | 0.48 – 1.43 | 0.510 |
| Alcohol intake: same vs never | 0.71 | 0.34 – 1.46 | 0.352 | 1.38 | 0.51 – 3.73 | 0.519 | 0.64 | 0.36 – 1.14 | 0.134 |
| Alcohol intake: increased vs never | 0.88 | 0.38 – 2.02 | 0.767 | 1.25 | 0.41 – 4.10 | 0.703 | 0.85 | 0.43 – 1.71 | 0.656 |
| COVID-19 symptoms: Yes vs No | 1.40 | 0.81 – 2.42 | 0.224 | 3.09 | 1.29 – 8.71 | **0.019** | 1.56 | 0.99 – 2.49 | 0.059 |
| Time spent outside: Yes vs No | 0.67 | 0.35 – 1.28 | 0.223 | 0.95 | 0.35 – 2.30 | 0.908 | 0.95 | 0.56 – 1.63 | 0.860 |
| Increased time using phone: Yes vs No | 1.27 | 0.63 – 2.65 | 0.506 | 0.65 | 0.27 – 1.46 | 0.318 | 1.22 | 0.72 – 2.05 | 0.458 |
| Sleep quality: same vs worsened | 0.41 | 0.21 – 0.76 | **0.005** | 0.55 | 0.27 – 1.14 | 0.108 | 0.72 | 0.45 – 1.14 | 0.159 |
| Sleep quality: improved vs worsened | 1.19 | 0.41 – 3.39 | 0.749 | 0.31 | 0.10 – 0.99 | **0.049** | 0.93 | 0.40 – 2.19 | 0.858 |

Table S2. Odds ratios (OR), 95% Confidence Intervals (CI) and p-values of the marginal effects and the interactions between each variable included in the regression models and sex.

|  | **Digestive disorders** | | **Headache** | | **COVID-19 fear** | |
| --- | --- | --- | --- | --- | --- | --- |
|  | OR (95%CI) | p | OR (95%CI) | p | OR (95%CI) | p |
| Relatives COVID-19 positives, with symptoms or dead: Female | 1.036 (0.833, 1.288) | 0.753 | 1.457 (1.084, 1.959) | 0.013 | 1.181 (0.983, 1.418) | 0.075 |
| Relatives COVID-19 positives, with symptoms or dead: Male | 1.511 (1.072, 2.131) | 0.019 | 1.106 (0.832, 1.469) | 0.490 | 0.945 (0.765, 1.167) | 0.597 |
| Relatives COVID-19 positives, with symptoms or dead: Female vs Male | 1.459 (0.982, 2.168) | 0.061 | 0.759 (0.511, 1.126) | 0.171 | 0.8 (0.609, 1.05) | 0.108 |
| Lockdown conditions (garden, cohabitants, rooms): Female | 1.051 (0.743, 1.487) | 0.778 | 0.872 (0.504, 1.509) | 0.625 | 1.115 (0.828, 1.501) | 0.475 |
| Lockdown conditions (garden, cohabitants, rooms): Male | 0.693 (0.428, 1.121) | 0.135 | 1.01 (0.593, 1.721) | 0.971 | 0.751 (0.515, 1.094) | 0.135 |
| Lockdown conditions (garden, cohabitants, rooms): Female vs Male | 0.659 (0.364, 1.192) | 0.168 | 1.158 (0.543, 2.47) | 0.704 | 0.673 (0.417, 1.087) | 0.105 |
| TV usage: Female | 1.148 (0.87, 1.515) | 0.330 | 1.484 (0.87, 2.533) | 0.148 | 0.992 (0.781, 1.26) | 0.950 |
| TV usage: Male | 0.858 (0.542, 1.358) | 0.513 | 1.279 (0.812, 2.017) | 0.289 | 1.253 (0.924, 1.7) | 0.147 |
| TV usage: Female vs Male | 0.747 (0.44, 1.269) | 0.281 | 0.862 (0.434, 1.714) | 0.672 | 1.263 (0.864, 1.845) | 0.227 |
| Mnemonic difficulties and performance reduction: Female | 1.242 (0.882, 1.749) | 0.214 | 1.123 (0.665, 1.896) | 0.665 | 1.01 (0.757, 1.348) | 0.946 |
| Mnemonic difficulties and performance reduction: Male | 1.37 (0.823, 2.279) | 0.226 | 1.191 (0.724, 1.96) | 0.491 | 1.013 (0.716, 1.434) | 0.942 |
| Mnemonic difficulties and performance reduction: Female vs Male | 1.103 (0.605, 2.011) | 0.750 | 1.061 (0.527, 2.135) | 0.868 | 1.003 (0.646, 1.556) | 0.990 |
| Increased physical activity, lost weight, improved nutrition: Female | 0.587 (0.405, 0.85) | 0.005 | 1.12 (0.647, 1.938) | 0.686 | 0.989 (0.722, 1.355) | 0.947 |
| Increased physical activity, lost weight, improved nutrition: Male | 0.453 (0.241, 0.853) | 0.014 | 0.824 (0.457, 1.488) | 0.522 | 0.727 (0.474, 1.114) | 0.143 |
| Increased physical activity, lost weight, improved nutrition: Female vs Male | 0.772 (0.378, 1.575) | 0.476 | 0.736 (0.339, 1.601) | 0.440 | 0.735 (0.437, 1.233) | 0.243 |
| Having been positive to SARS-CoV-2: Female | 0.842 (0.393, 1.801) | 0.930 | 1.782 (0.498, 6.372) | 0.625 | 1.425 (0.745, 2.727) | 0.473 |
| Having been positive to SARS-CoV-2: Male | 2.254 (0.727, 6.991) | 0.237 | 0.893 (0.29, 2.746) | 0.993 | 1.154 (0.52, 2.562) | 0.963 |
| Having been positive to SARS-CoV-2: Female vs Male | 2.678 (0.682, 10.523) | 0.236 | 0.501 (0.089, 2.828) | 0.713 | 0.81 (0.287, 2.286) | 0.948 |
| Social isolation: Female | 1.287 (0.574, 2.884) | 0.839 | 1.776 (0.53, 5.947) | 0.589 | 1.118 (0.563, 2.22) | 0.972 |
| Social isolation: Male | 0.799 (0.232, 2.755) | 0.963 | 1.281 (0.396, 4.146) | 0.943 | 1.36 (0.587, 3.15) | 0.765 |
| Social isolation: Female vs Male | 0.621 (0.142, 2.718) | 0.825 | 0.721 (0.137, 3.8) | 0.953 | 1.216 (0.418, 3.536) | 0.961 |
| Decreased vs never smoking: Female | 0.582 (0.106, 3.178) | 0.986 | 0.253 (0.037, 1.728) | 0.357 | 1.281 (0.323, 5.085) | 1.000 |
| Same vs never smoking: Female | 0.511 (0.101, 2.576) | 0.926 | 0.275 (0.033, 2.287) | 0.580 | 1.216 (0.322, 4.59) | 1.000 |
| Increased vs never smoking: Female | 2.576 (0.61, 10.876) | 0.476 | 1.696 (0.077, 37.425) | 1.000 | 0.991 (0.314, 3.129) | 1.000 |
| Decreased vs never smoking: Male | 2.14 (0.236, 19.389) | 0.976 | 0.619 (0.065, 5.922) | 0.999 | 1.395 (0.263, 7.388) | 1.000 |
| Same vs never smoking: Male | 3.116 (0.588, 16.503) | 0.423 | 0.46 (0.085, 2.498) | 0.872 | 0.964 (0.267, 3.488) | 1.000 |
| Increased vs never smoking: Male | 1.51 (0.217, 10.527) | 0.999 | 1.049 (0.136, 8.094) | 1.000 | 0.828 (0.217, 3.157) | 1.000 |
| Decreased vs never smoking: Female vs Male | 3.681 (0.234, 57.81) | 0.851 | 2.449 (0.135, 44.364) | 0.989 | 1.089 (0.128, 9.278) | 1.000 |
| Same vs never smoking: Female vs Male | 6.099 (0.623, 59.719) | 0.228 | 1.672 (0.118, 23.696) | 1.000 | 0.793 (0.129, 4.896) | 1.000 |
| Increased vs never smoking: Female vs Male | 0.586 (0.054, 6.392) | 0.999 | 0.619 (0.015, 25.347) | 1.000 | 0.836 (0.144, 4.863) | 1.000 |
| Decreased vs never alcohol drinking: Female | 1.148 (0.379, 3.479) | 1.000 | 0.51 (0.069, 3.798) | 0.980 | 1.103 (0.42, 2.896) | 1.000 |
| Same vs never alcohol drinking: Female | 0.826 (0.246, 2.777) | 1.000 | 0.617 (0.071, 5.342) | 0.999 | 0.697 (0.244, 1.993) | 0.977 |
| Increased vs never alcohol drinking: Female | 0.745 (0.18, 3.089) | 0.999 | 0.461 (0.037, 5.821) | 0.990 | 0.633 (0.183, 2.19) | 0.964 |
| Decreased vs never alcohol drinking: Male | 0.589 (0.1, 3.469) | 0.991 | 1.523 (0.279, 8.317) | 0.998 | 0.495 (0.142, 1.721) | 0.678 |
| Same vs never alcohol drinking: Male | 0.522 (0.089, 3.043) | 0.963 | 2.603 (0.417, 16.25) | 0.765 | 0.586 (0.168, 2.043) | 0.912 |
| Increased vs never alcohol drinking: Male | 1.167 (0.183, 7.466) | 1.000 | 2.491 (0.3, 20.656) | 0.908 | 1.423 (0.306, 6.618) | 0.999 |
| Decreased vs never alcohol drinking: Female vs Male | 0.513 (0.065, 4.054) | 0.985 | 2.984 (0.227, 39.185) | 0.916 | 0.448 (0.094, 2.145) | 0.784 |
| Same vs never alcohol drinking: Female vs Male | 0.632 (0.078, 5.145) | 0.999 | 4.221 (0.27, 65.974) | 0.762 | 0.841 (0.169, 4.176) | 1.000 |
| Increased vs never alcohol drinking: Female vs Male | 1.566 (0.158, 15.566) | 1.000 | 5.399 (0.204, 142.749) | 0.779 | 2.247 (0.318, 15.873) | 0.927 |
| COVID-19 symptoms: Female | 1.018 (0.464, 2.233) | 1.000 | 10.625 (0.85, 132.842) | 0.075 | 1.838 (0.911, 3.708) | 0.111 |
| COVID-19 symptoms: Male | 2.989 (0.917, 9.743) | 0.079 | 1.558 (0.391, 6.212) | 0.828 | 1.176 (0.473, 2.923) | 0.964 |
| COVID-19 symptoms: Female vs Male | 2.935 (0.715, 12.047) | 0.192 | 0.147 (0.008, 2.606) | 0.298 | 0.64 (0.205, 1.992) | 0.722 |
| Time spent outside: Female | 0.815 (0.318, 2.089) |  |  | 1.000 | 1.14 (0.495, 2.627) | 0.975 |
| Time spent outside: Male | 0.459 (0.128, 1.652) |  |  | 0.252 | 0.739 (0.274, 1.995) | 0.849 |
| Time spent outside: Female vs Male | 0.564 (0.119, 2.675) |  |  | 1.000 | 0.648 (0.182, 2.306) | 0.800 |
| Increased time using phone: Female | 1.11 (0.412, 2.991) | 0.992 | 0.978 (0.219, 4.371) | 1.000 | 1.426 (0.624, 3.259) | 0.664 |
| Increased time using phone: Male | 2.005 (0.29, 13.877) | 0.774 | 0.474 (0.117, 1.916) | 0.492 | 0.975 (0.364, 2.615) | 1.000 |
| Increased time using phone: Female vs Male | 1.806 (0.206, 15.87) | 0.887 | 0.485 (0.061, 3.831) | 0.787 | 0.684 (0.19, 2.462) | 0.858 |
| Same vs worsened sleep quality: Female | 0.442 (0.165, 1.184) | 0.163 | 0.341 (0.089, 1.31) | 0.195 | 0.669 (0.3, 1.49) | 0.710 |
| Improved vs worsened sleep quality: Female | 1.41 (0.282, 7.042) | 0.994 | 0.572 (0.051, 6.424) | 0.991 | 1.124 (0.282, 4.473) | 1.000 |
| Same vs worsened sleep quality: Male | 0.337 (0.069, 1.649) | 0.360 | 0.827 (0.219, 3.119) | 0.999 | 0.772 (0.309, 1.932) | 0.975 |
| Improved vs worsened sleep quality: Male | 0.656 (0.027, 16.037) | 1.000 | 0.129 (0.012, 1.435) | 0.142 | 0.615 (0.089, 4.252) | 0.986 |
| Same vs worsened sleep quality: Female vs Male | 0.764 (0.121, 4.822) | 0.999 | 2.426 (0.392, 14.996) | 0.739 | 1.155 (0.353, 3.779) | 1.000 |
| Improved vs worsened sleep quality: Female vs Male | 0.465 (0.014, 15.91) | 0.994 | 0.225 (0.008, 6.434) | 0.810 | 0.547 (0.053, 5.689) | 0.984 |

Note: In the association with headache, the interaction term between time spent outside and sex was omitted since there were no females that did not spend any time outside without headache.
